# Adaptive sequence evolution in a color gene involved in the formation of the characteristic egg-dummies of male haplochromine cichlid fishes

**DOI:** 10.1186/1741-7007-5-51

**Published:** 2007-11-15

**Authors:** Walter Salzburger, Ingo Braasch, Axel Meyer

**Affiliations:** 1 Lehrstuhl für Zoologie und Evolutionsbiologie, Department of Biology, University Konstanz, 78457 Konstanz, Germany; 2 Department of Ecology and Evolution, University of Lausanne, 1015 Lausanne, Switzerland; 3 Zoologisches Institut der Universität Basel, Evolutionsbiologie, Universität Basel, 4051 Basel, Switzerland; 4 Physiological Chemistry I, University of Würzburg, 97074 Würzburg, Germany

## Abstract

**Background:**

The exceptionally diverse species flocks of cichlid fishes in East Africa are prime examples of parallel adaptive radiations. About 80% of East Africa's more than 1 800 endemic cichlid species, and all species of the flocks of Lakes Victoria and Malawi, belong to a particularly rapidly evolving lineage, the haplochromines. One characteristic feature of the haplochromines is their possession of egg-dummies on the males' anal fins. These egg-spots mimic real eggs and play an important role in the mating system of these maternal mouthbrooding fish.

**Results:**

Here, we show that the egg-spots of haplochromines are made up of yellow pigment cells, xanthophores, and that a gene coding for a type III receptor tyrosine kinase, *colony-stimulating factor 1 receptor a *(*csf1ra*), is expressed in egg-spot tissue. Molecular evolutionary analyses reveal that the extracellular ligand-binding and receptor-interacting domain of *csf1ra *underwent adaptive sequence evolution in the ancestral lineage of the haplochromines, coinciding with the emergence of egg-dummies. We also find that *csf1ra *is expressed in the egg-dummies of a distantly related cichlid species, the ectodine cichlid *Ophthalmotilapia ventralis*, in which markings with similar functions evolved on the pelvic fin in convergence to those of the haplochromines.

**Conclusion:**

We conclude that modifications of existing signal transduction mechanisms might have evolved in the haplochromine lineage in association with the origination of anal fin egg-dummies. That positive selection has acted during the evolution of a color gene that seems to be involved in the morphogenesis of a sexually selected trait, the egg-dummies, highlights the importance of further investigations of the comparative genomic basis of the phenotypic diversification of cichlid fishes.

## Background

**Figure 1:**
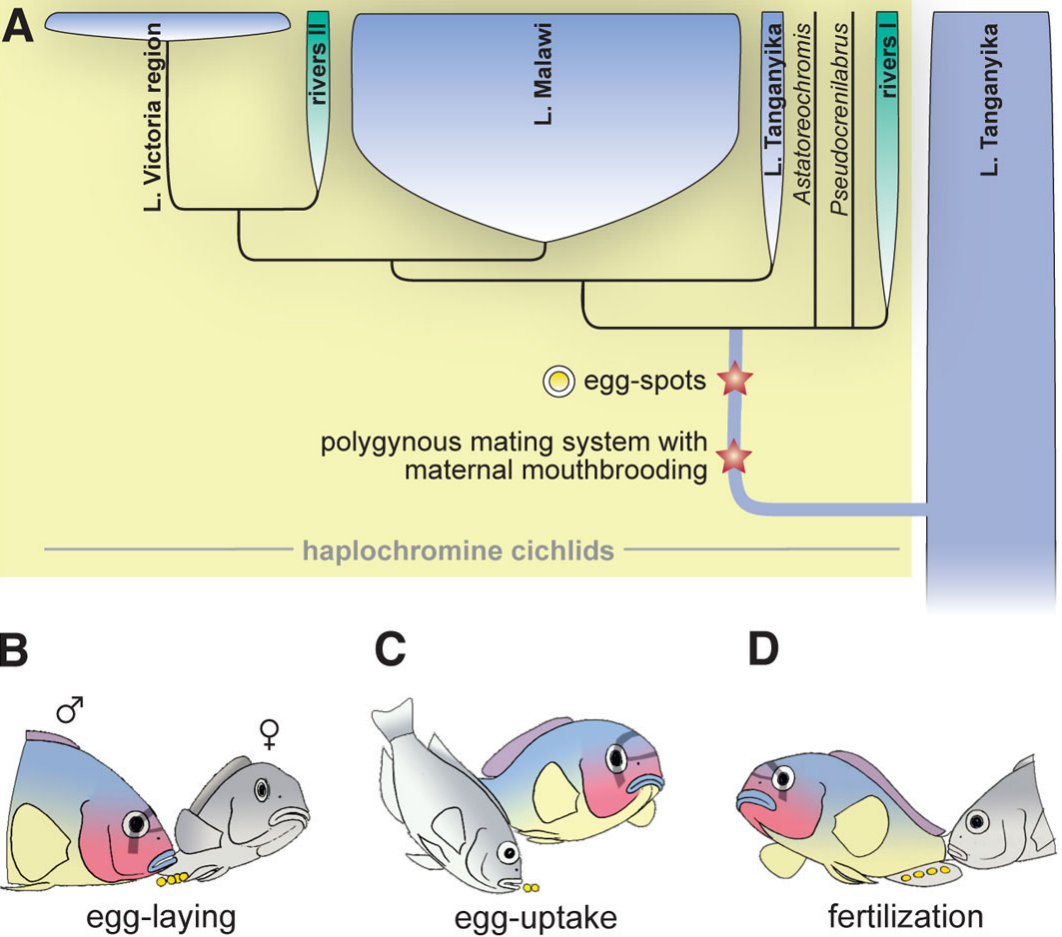
**Haplochromine evolution in Africa**. (a) Consensus phylogeny of East African cichlids based on mitochondrial [7] and nuclear DNA markers (this study). The haplochromines are derived from the much older cichlid species assemblage of Lake Tanganyika. In only about two million years of evolution, the haplochromines gave rise to spectacular adaptive radiations in Lakes Tanganyika (~30 species), Malawi (up to 1000 species) and Victoria (~500 species), as well as in other lakes and rivers in Africa (up to 200 species) [1,3,7–9]. Evolutionary innovations are mapped onto the phylogeny [7]. Note that mouthbrooding evolved several times during cichlid evolution, but only the haplochromines show a characteristic polygynous maternal mouthbrooding system with egg-spots as sexual advertisement [7,11–14,17]. (b-d) The egg-spots on the anal fins of male haplochromines play a crucial role in the haplochromines' reproductive system. Three main phases of the spawning cycle are shown: (b) the female lays a small batch of eggs in the territory of a male. (c) The female takes up the eggs into her mouth. (d) The male presents its conspicuous egg-spots on his anal fin. The female is attracted by these egg-dummies and tries to pick them up, thereby bringing her mouth close to the male's genital opening, through which sperm is released.

Cichlid fishes in general, and the species flocks of cichlids in the East African Great Lakes in particular, are premier examples of animal adaptive radiations [1-4]. The cichlid species flocks of Lakes Victoria, Malawi and Tanganyika are an excellent model system to study the genetic basis of organismal diversity, because of their enormous phenotypic diversity, their species-richness, their close relatedness and the repeated occurrences of evolutionary parallelisms (see e.g., [2,3,5,6]). About 80% of East Africa's endemic cichlid species, and all species of the species flocks of Lakes Victoria and Malawi (plus one lineage from Lake Tanganyika) belong to a particularly successful lineage of cichlids, the haplochromines [7-9]. Only a few synapomorphies of the haplochromines have been identified to date. All taxa appear to have a similar type of upper pharyngeal bones [10]. Two additional characteristic features of the haplochromines are a polygynous and/or polygynandrous mating system and maternal mouthbrooding, with only females incubating the eggs in their buccal cavities, as well as 'egg-dummies' (*ocelli*) in the form of ovoid markings on the anal fins of males that mimic real eggs in size, color and shape [7,11-16] (Figure 1).

The males' yellow to orange egg-spots play a crucial role in the haplochromines' mating system in that they serve as intra-specific sexual advertisement to attract females and to maximize breeding success [11,16,17], possibly as a response to predation pressure from con-specifics [18]: A female with ripe eggs initiates breeding by approaching a territorial courting male, which responds with a lateral display in the form of quivering and fluttering the unpaired anal, caudal and dorsal fins, thereby exposing its bright coloration. The female lays a small batch of eggs (Figure 1b), circles around, and picks them up in her mouth (Figure 1c) before fertilization. Attracted by the egg-spots on the males' anal fin, the female attempts to ingest these dummies and brings her mouth into close proximity to the males' genital opening – this is when the male discharges sperm (Figure 1d; see Additional file 1). In this way, the fertilization of the eggs takes place within the females' mouth [11,12,14,15]. We note that some variations from this general procedure have been reported. For example, in some species the eggs are fertilized before being picked up by the female [19]. Haplochromine females were shown to prefer males with egg-spots over males where the dummies had been removed [17] or manipulated experimentally [20], and it has been suggested that sexual selection on egg-spot morphology could lead to speciation [21].

Haplochromine egg-dummies begin to form in the juvenile stage of males and start out from the edge of the anal fin [12], though they only begin to brighten when the young males reach sexual maturity. Haplochromine species differ greatly in egg-spot number, arrangement and morphology. Anal fin egg-dummies are, however, not an exclusive male characteristic. In some species, females also show ovoid markings on their anal fins, but these are typically much less conspicuous than the egg-spots of males. A typical haplochromine egg-dummy is characterized by a conspicuous yellow to reddish central area and a more or less transparent outer ring [11,12,14]. This type of egg-dummy is found in most riverine and rock-dwelling haplochromines, while other species, such as more ancestral riverine taxa or pelagic and sand-dwelling species in Lake Malawi, sometimes show a more amorphic blotch pattern. Additionally, a small number of extant haplochromines lack egg-spots entirely, and it has been suggested that they have lost their dummies secondarily [7].

Yellow blotches that also act as egg-dummies are also known from a few species of the Lake Tanganyika tribe Ectodini, which are only distantly related to the haplochromines (see e.g., [22]). However, the ectodines' egg-dummies are morphologically less complex than those of the haplochromines and they are found on the paired pelvic fins instead of the unpaired anal fins as in haplochromines. Finally, some mouthbrooding Tilapia species have evolved filamentous arborescent appendages at their genital papillae, so-called genital tassels, which act as egg-dummies [11,14].

Here, we report on the identification of a gene that is likely to play a role in the development of the yellow xanthophores in the haplochromines' egg-spots. We hypothesized that a previously isolated xanthophore-related color gene might be involved in the formation of xanthophores in the egg-spots: The *colony-stimulating factor 1 receptor a *(*csf1ra*) gene, coding for a type III receptor tyrosine kinase [23,24], is known to be expressed by cells of the xanthophore lineage in zebrafish; it is essential for recruiting xanthophores from their precursors, and it is indirectly involved in the organization of the dark melanophores [25,26]. Zebrafish mutants for *csf1ra *(*panther*) exhibit disrupted stripe patterns and lack xanthophores [25], and it has been shown that some species in the genus *Danio *vary in their *csf1ra *pathway during pigment pattern formation [27]. *csf1ra *is, thus, not only an important marker for the xanthophore lineage in zebrafish, but is also involved in xanthophore development and, possibly, color patterning. In addition to its role in body coloration, *csf1ra *is expressed in the macrophage and osteoclast cell lineage in zebrafish [25]. We performed RT-PCR and *in situ *hybridization experiments in several haplochromine cichlid species in order to confirm the expression of *csf1ra *in the males' egg-spots. For detailed evolutionary analyses, we determined the DNA sequence of this gene locus in 19 East African cichlid species and tested for the signature of adaptive evolution in the haplochromine lineage. Finally, we also tested for *csf1ra *expression in the ectodine species *Ophthalmotilapia ventralis*, which has egg-dummies at the end of the paired pelvic fins.

## Results

### Gene expression assays

**Figure 2:**
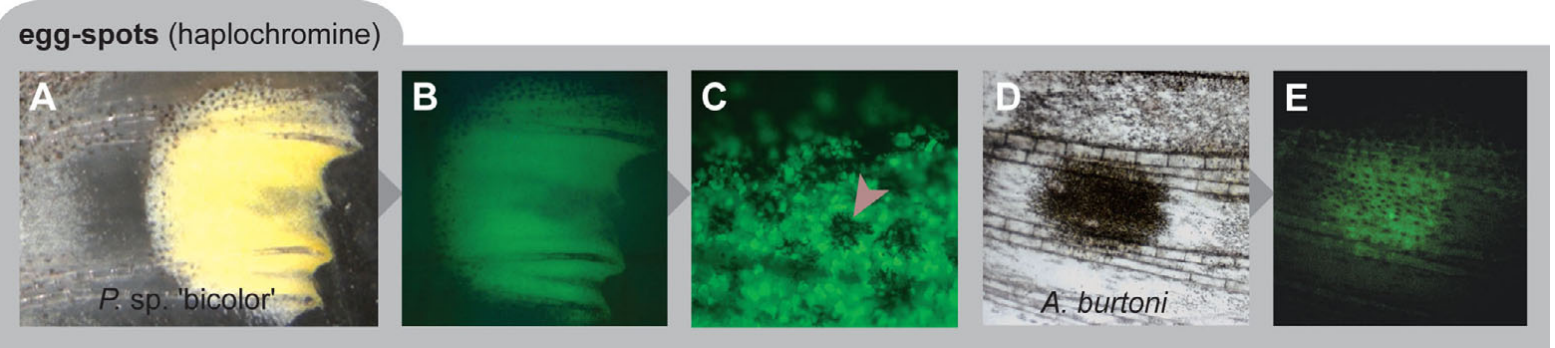
**The haplochromines' egg-spots are made up of xanthophores**. (a-c) Photograph (a) and fluorescence image (b, c) of the egg-spot of *Pseudotropheus *sp 'bicolor'. The yellow inner ring of the egg-spot of *P*. sp. 'bicolor' consists of xanthophores, the pteridine pigments of which fluoresce at high pH. Melanophores are also present in the border area around the transparent outer ring (a melanophore is marked by an arrowhead). (d, e) Egg-spot of *Astatotilapia burtoni*; bright field (d) and fluorescence image (e).

**Figure 3:**
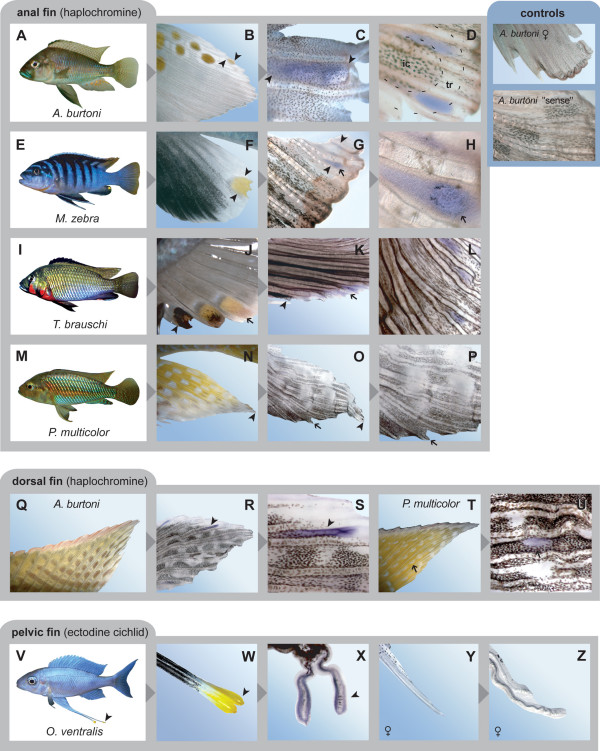
***In situ *hybridization experiments showing *csf1ra *expression in cichlids**. (a-d) In male *Astatotilapia burtoni *(a), which typically have more than 10 egg-spots arranged into two or more rows (b), we could detect *csf1ra *expression in the forming (c) as well as in the growing egg-dummies (d; the position of the yellowish inner circle, *ic*, and the transparent outer ring, *tr*, is indicated). Note that the yellow pigments of the egg-spots are removed during tissue processing. (e-h) In male *Pseudotropheus *sp. 'bicolor' (e), for which we used a morph with only one egg-spot (f), *csf1ra *expression was detected throughout the entire egg-spot (g), and it appears that the dark melanophores arrange around the center of *csf1ra *expression (h), just as reported for zebrafish, where *csf1ra *is indirectly also responsible for melanophore organization [25]. (i-l) In male *Thoracochromis brauschi *(i), *csf1ra *is expressed in the yellow blotches of the anal fin (j, k), and also in the pearly spots ('*Perlfleckmuster*') on the posterior part of the anal fin (l). (m-p) In the basal riverine haplochromine *Pseudocrenilabrus multicolor *(m), the male anal fins do not exhibit egg-spot like blotches (n) and no *csf1ra *expression could be detected either (o, p). (q-u) *csf1ra *is expressed in the yellowish/orange areas of the pearly spot pattern on the dorsal fins of male *A. burtoni *(q-s) and *P. multicolor *(t, u). (v-z) *csf1ra *is expressed in the egg-dummies on the tassels at the tips of the conspicuously elongated pelvic fins of the ectodine cichlid *Ophthalmotilapia ventralis *(v-x). Female fins of *O. ventralis *(y) do not show *csf1ra *expression (z). The control experiments show that *csf1ra *is not expressed in female anal fins or in the "sense" control. Arrowheads refer to identical points in sequential images.

By applying a fluorescence-based detection method, we first confirmed that the yellowish center of the haplochromines' egg-spots consists of a particular class of pigment cells, the xanthophores (Figure 2). The subsequent RT-PCR experiments from egg-spot tissue of *A. burtoni *were positive for the xanthophore-related color gene *csf1ra*. This was further confirmed by *in situ *hybridization experiments documenting *csf1ra *expression in the males' egg-dummies in all tested haplochromine species that displayed these ovoid markings (Figure 3a–k). In *Astatotilapia burtoni *males, which have up to a dozen egg-spots that are organized in two or more rows, we detected *csf1ra *expression in the developmentally younger and still growing egg-spots in proximity to the edge of the anal fin (Figure 3a–d), which is exactly where new egg-dummies begin to form [12]. Males of the *Pseudotropheus sp*. 'bicolor' population used for this study show a single egg-spot only, in which *csf1ra *is also expressed (Figure 3e–h). Similarly, *csf1ra *expression was detected in the yellow markings of *Thoracochromis brauschi *(Figure 3i–k), a member of a basal riverine haplochromine lineage [7] with rather unstructured yellow blotches. The egg-spot free anal fins of *P. multicolor *[7,12], however, do not show *csf1ra *expression (Figure 3m–p). This is not due to the use of a heterologous probe designed on the basis of *csf1ra *from *A. burtoni*, as other patterns in *P. multicolor *skin revealed *csf1ra *positive results (see below). Female anal fins and negative controls did not show any labeling (Figure 3). Furthermore, we determined *csf1ra *expression in the pearly spot pattern in the dorsal fin of *A. burtoni *and *P. multicolor *(Figure 3q–u) and in the posterior part of the anal fin of *T. brauschi *(Figure 3l). Expression of *csf1ra *was also detected in the tassels at the tips of the conspicuously elongated paired ventral fins of males of the non-haplochromine Tanganyikan species *Ophthalmotilapia ventralis*, where these yellow markings also function as egg-dummies [14] (Figure 3v–x), but not in the similarly elongated female ventral fins of *O. ventralis *that lack egg-dummies (Figure 3y, z).

### Phylogenetic and molecular evolutionary analyses of the *csf1ra *locus

**Figure 4:**
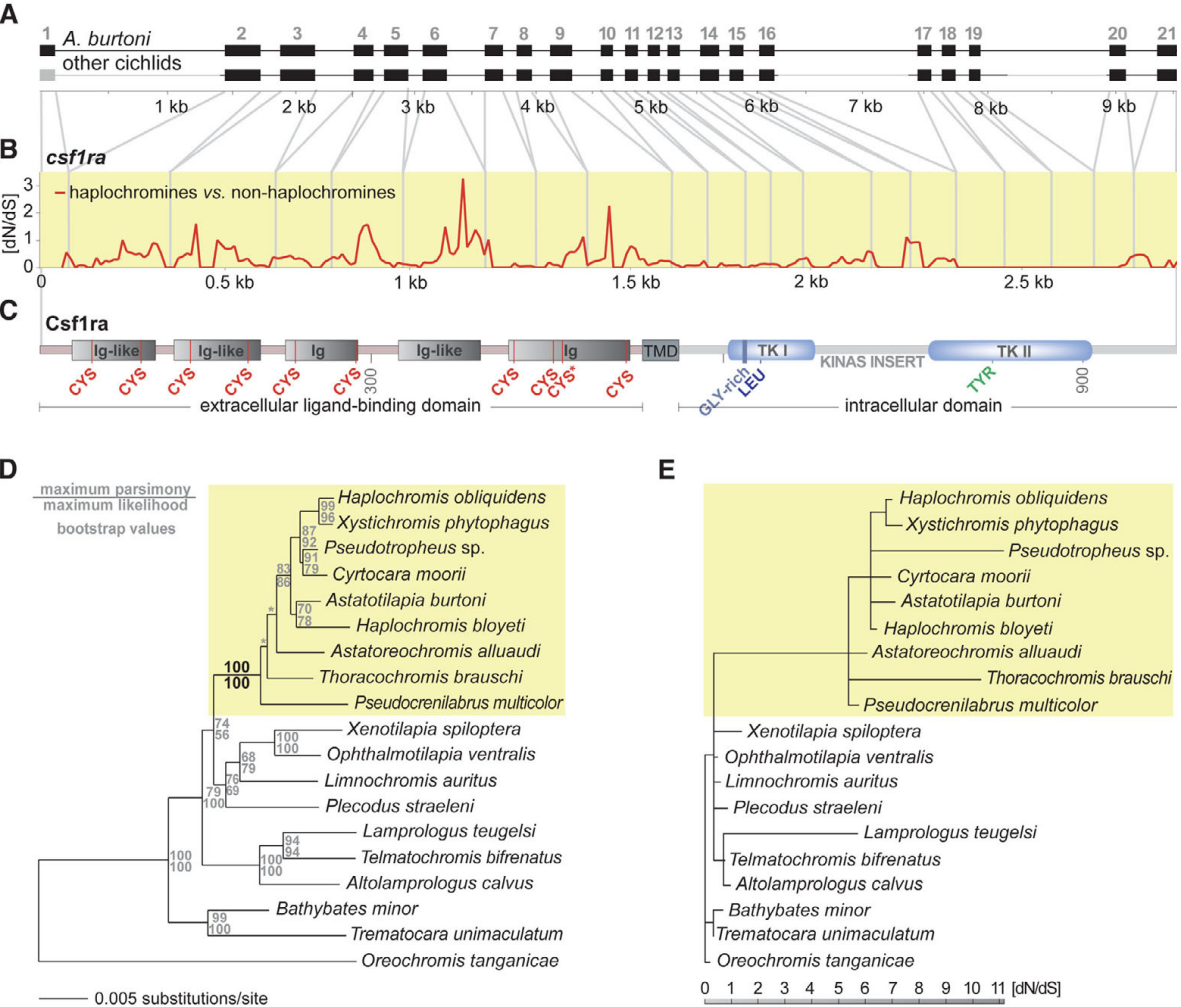
**Molecular evolutionary analyses of the *csf1ra *locus**. (a) The entire *csf1ra *gene locus was obtained from 19 cichlid species (exon 1 and the relatively large introns 1, 16, and 19, which are shown in gray, were not sequenced). (b) *d*N/*d*S ratio of haplochromines compared to non-haplochromine cichlids as revealed from a sliding window analysis with DNASP. (c) Schematic representation of the structure of the Csf1ra protein. The gene consists of an extracellular ligand-binding domain containing five immunoglobulin-like (Ig-like) domains with conserved cysteines (CYS), a transmembrane domain (TMD), and an intracellular partition that contains two tyrosine kinase (TK) domains interrupted by a kinase insert domain. In the tyrosine kinase I domain (TK I) a glycine-rich region (GLY-rich) and a conserved leucine (LEU) is found, the tyrosine kinase II domain (TK II) contains a conserved tyrosine (TYR). The asterisk indicates that in one species, *X. phytophagus*, the otherwise conserved cysteine at amino acid position 450 has been replaced by a tryptophan. (d) Maximum likelihood phylogeny based on more than 4100 bp of non-coding sequences of the *csf1ra *gene locus corroborating the monophyly of the haplochromines (yellow box) and the ancestral position of *A. alluaudi*, *P. multicolor*, and *T. brauschi *[7]. The asterisk indicates relatively short branches not supported by high bootstrap values or a Shimodaira-Hasegawa test. (e) Branch-scaled tree showing the *d*N/*d*S rates reconstructed with HyPhy. The only internal branch with a *d*N/*d*S > 1 is the one representing the common ancestor of the haplochromines (yellow box).

We sequenced more than 7 000 bp of the *csf1ra *locus of 19 cichlid species representing the phylogenetic diversity of cichlids in East Africa (Figure 4a–c). Our phylogenetic analyses, based on the intron sequences of the *csf1ra *locus (> 4 000 bp; Figure 4d), confirmed previous phylogenetic hypotheses by placing the representatives of the Bathybatini and Trematocarini as sister group to a clade comprised by the substrate spawning lamprologines (represented by *A*. *calvus*, *L. teugelsi*, and *T. bifrenatus*) and the East African mouthbrooding cichlids (remaining taxa) [7,22,28]. The haplochromines (yellow box in Figure 4d) form a strongly supported monophyletic clade within these mouthbrooders, with *P. multicolor, T. brauschi *and *A. alluaudi *branching off ancestrally to the exceptionally species-rich 'modern haplochromines' represented by *A. burtoni, C. moorii, H. bloyeti, H. obliquidens, P*. sp., and *X. phytophagus *[7]. The bootstrap values were generally high, except for the branches connecting the four main lineages of haplochromines. Just as in our previous molecular phylogenetic analysis [7], the Shimodaira-Hasegawa test did not favor a particular phylogenetic hypothesis with respect to the interrelationship of the four main lineages of haplochromines.

The ancestral state reconstructions using a consensus tree on the basis of this newly-generated phylogenetic hypothesis (Figure 4d) and a previously published mitochondrial phylogeny [7] revealed that both the particular mating system and the egg-dummies on male anal fins are likely to have evolved only once in the ancestral lineage of the haplochromines (see Figure 1a). Thus, the lack of egg-spots in a few haplochromine species is most likely due to secondary loss.

We then tested for the existence of a signal of adaptive sequence evolution in the coding region of *csf1ra*. This gene consists of 21 exons with a combined length of 2 928 bp, and is made up of a cysteine-rich extracellular ligand-binding domain composed of five immunoglobulin-like domains, a transmembrane domain, and an intracellular domain with two separate tyrosine kinase domains [23,29] (Figure 4a, c). A sliding window analysis with DNASP [30] uncovered several sections in *csf1ra *of haplochromines with a *d*N/*d*S ratio greater than one (Figure 4b), which would indicate that positive selection has acted to shape the protein. These regions were primarily located in the first 1 551 bp of the gene (amino acid positions 1 to 517) corresponding to the extracellular ligand-binding domain of Csf1ra. In this domain, there are in total more non-synonymous substitutions than synonymous ones in the haplochromines (see amino acid alignment in Additional file 2), but not in the more basal cichlid lineages that are without anal fin egg-dummies. The maximum likelihood reconstructions of *d*N/*d*S ratios in this domain of *csf1ra *revealed that the only internal branch with a *d*N/*d*S >> 1 is the one representing the common ancestor of the haplochromines (*d*N/*d*S > 5; Figure 4e). Such a signal of adaptive sequence evolution specific to the haplochromine lineage could not be detected in a segment of the extracellular domain of *kita*, another type III receptor tyrosine kinase with a known function in pigment patterning [31], which we have sequenced in the 19 cichlid species as a control. Also, in *kita *there was no indication that the segment of the extracellular domain would have evolved under a different selection regime as compared to a segment in the intracellular domain (Additional file 3).

We also analyzed a genomic region of approximately 1 000 bp upstream of *csf1ra *in the 19 cichlid species, to investigate sequence differences in putative gene regulatory regions. The only mutation common to all haplochromines, distinguishing them from the more ancestral and less species-rich cichlid lineages, involves a mutation in a putative transcription factor-binding site, a TATA box, about 130 bp upstream of *csf1ra*.

## Discussion

### *csf1ra *is expressed in egg-dummies of haplochromine cichlids

Egg-dummies on the male anal fins play an important role during the breeding behavior of the female mouthbrooding haplochromines (see e.g., [7,13,14]). Phylogenetic and character state reconstructions corroborate that these egg-dummies evolved only once in the ancestral lineage of haplochromines [7] (Figure 1a), and that the absence of ovoid markings on the anal fins of some haplochromine species is due to secondary loss, e.g. because of reasons of camouflage or as an adaptation to the deep-water habitat and visual environment where these markings would not be easily visible.

Haplochromine egg-spots vary in size, shape, number and arrangement in different species. Figure 3 provides some examples of the diversity of egg-spots found in haplochromines. A typical egg-dummy consists of a conspicuous yellow to reddish central area and a more or less transparent outer ring [11,12,14] (see Figure 3b, f), although a number of species only show amorphic blotches (see e.g., Figure 3j). The brightly colored inner circle is, as we have shown (Figure 2), made up of xanthophores. Yellowish, orange, or reddish xanthophores also occur in cichlid fins other than the anal fin (see e.g., Figure 3q–u) and in skin tissue, where substantial differences in densities – depending on coloration patterns – can be found (Clabaut, Salzburger and Meyer, unpublished results). However, nowhere (not even in yellow colored fish) could we identify a higher density of xanthophores than in the egg-dummies of haplochromine males.

We applied a candidate gene approach in order to test whether the previously isolated xanthophore-related color gene *csf1ra *[25,26] is expressed in haplochromine egg-spots. Our *in situ *hybridization experiments indeed corroborate *csf1ra *expression in the egg-dummies of all tested haplochromine species (Figure 3). We detected *csf1ra *expression in the younger and still growing egg-spots of *A. burtoni *(Figure 3a–d), in the single egg-dummy of *P. sp*. 'bicolor' (Figure 3e–h), and in the relatively unstructured male anal fin blotches of *T. brauschi *(Figure 3i–k), which is a member of an ancestral riverine haplochromine clade. The simple orange blotches of *T. brauschi *(Figure 3j) again illustrate (see above) that not all types of egg-dummies show a clear-cut separation into a brightly colored inner circle and a more or less transparent outer ring. In this specific case, it could, however, be argued that because of the phylogenetic position of this species, the undifferentiated spots of *T. brauschi *represent an intermediate character state in the evolution of egg-dummies. That *csf1ra *is expressed in egg-spots of younger males and the smaller and still developing egg-spots of adult males might indicate that *csf1ra *is required for xanthophore recruitment from pigment cell precursors during egg-spot formation, just as has been reported for the formation of stripes in zebrafish [25,26]. This would need to be tested in future experiments.

In view of the fact that *csf1ra *is expressed in both patterns, the comparative *in situ *hybridization experiments also seem to support the earlier suggestion that egg-spots of haplochromines are likely to be derived from the '*Perlfleckmuster' *found in unpaired fins of haplochromines and many other cichlid species [11,12]. Specifically, we show that *csf1ra *is expressed in pearly spots on dorsal fins of *A. burtoni *and *P. multicolor *(Figure 3q–u). As such pearly spots are also found on anal fins of some (ancestral) haplochromine lineages (see Figure 3l for *csf1ra *expression in the pearly spots on the posterior part of the anal fin of *T. brauschi*), a co-option of at least some aspects of the molecular basis of the pearly blotch pattern for the formation of egg-dummies appears likely.

### Adaptive sequence evolution in the extracellular domain of *csf1ra *in haplochromines

In order to investigate the molecular evolutionary history of *csf1ra *we sequenced large fractions of the locus in 19 representative cichlid species (Figure 4a). The comparison of haplochromine with non-haplochromine species revealed that several regions in *csf1ra *show a *d*N/*d*S ratio greater than one (Figure 4b), indicating that positive selection (adaptive sequence evolution) has acted to change the protein in haplochromines. Positive Darwinian selection often only acts on particular domains of a gene, whereas other sections remain subject to purifying selection. Also, adaptive evolution is expected to act only at particular times during the evolution of a lineage. In our case, we found that the regions showing a *d*N/*d*S > 1 are located in the part of the gene that encodes the extracellular domain. The Csf1ra protein functions as membrane spanning cell surface receptor [29,32], and is characterized by a cysteine-rich extracellular ligand-binding domain composed of five immunoglobulin-like domains containing growth factor binding sites, a transmembrane domain, and two separate tyrosine kinase domains [23,29] (Figure 4c). A maximum likelihood reconstruction of *d*N/*d*S ratios revealed that the extracellular domain of *csf1ra *underwent adaptive evolution in the common ancestor of the haplochromines (Figure 4e) – simultaneous to when the egg-dummies are likely to have evolved. The occurrence of adaptive changes in the amino acid sequence in the ligand-binding portion of *csf1ra *(Figure 4e, Additional file 2) seems to suggest that novel modifications of existing signal transduction mechanisms evolved in the haplochromine lineage that were associated with the evolution of egg-spots, or, possibly, other color patterns involving xanthophores. Functional assays (see e.g., [33]) are now required to test whether the observed differences in the coding sequence of *csf1ra *have any effect on ligand-receptor interactions. Similarly, a more thorough comparative analysis of the upstream region of *csf1ra *is necessary to test the possibility that regulatory elements also underwent evolutionary changes in the ancestor of haplochromines, as is suggested by the observed haplochromine-specific mutation in a putative transcription factor binding site and the differential expression of *csf1ra *between haplochromine and non-haplochromine cichlids. In addition, *csf1ra *expression should also be investigated in other tissues and cell lineages. Although it is not apparent how e.g., macrophages or osteoclasts (in which *csf1ra *is also expressed [25]) could contribute to the evolutionary success of haplochromines but not of other cichlids, the remote possibility remains that the signal of adaptive sequence evolution is due to functions other than coloration.

To date, evidence for accelerated protein evolution in haplochromines has been found in the bone morphogenetic protein 4 (*bmp4*) that is hypothesized to be involved in jaw formation ([34]; see also [5,35]), in a color perception gene, the long wavelength-sensitive (LWS) opsin [36], and in a putative color gene, the F-box-WD-repeat *hagoromo *[37]. The finding of signatures of adaptive sequence evolution in a jaw-related gene, as well as in color and color-perception genes, seems to corroborate the hypothesis that both the particular architecture of the cichlids' jaw apparatus and the haplochromines' mating system are important traits that have contributed to the evolutionary success of cichlid fishes in general and of haplochromines in particular [7,13,14,38]. The adaptive advantage of the mating system of the haplochromines (with coloration and egg-spots as sexual advertisement) might be the facilitation of sexual selection through female choice (see e.g., [7,16,21]). Sexual selection has been suggested as a major cause for the explosive origin of new species of cichlids in species flocks [3,13,39,40].

### *csf1ra *is also expressed in egg-dummies of ectodine cichlids

One of the most fascinating aspects of cichlid evolution is the repeated occurrence of evolutionary parallelisms [1,41-43]. This has led to the question of whether natural selection alone is sufficient to produce parallel morphologies or whether a developmental or genetic bias has influenced the direction of diversification [6]. Because of their independent origin in at least two lineages of mouthbrooding cichlids (not counting the genital tassels of some Tilapia species), egg-dummies on cichlid fins are likely to represent another example of evolutionary parallelism in the adaptive radiations of cichlids in East Africa – in this case involving a rather complex ethomorphological trait.

The function of egg-dummies in mimicking eggs to attract females is known from haplochromines and also from ectodines such as *O. ventralis *[14] (Figure 3v). Nevertheless, the dummies of *O. ventralis *(and its congeners) and those of haplochromines are of independent evolutionary origin, and they show different degrees of complexity. Most importantly, the two kinds of egg-markings are found on different anatomical structures, leading to substantial differences in the spawning behavior. In male haplochromines, the often numerous egg-spots are situated on the anal fin and, hence, are in close proximity to the genital opening to which the female's mouth is supposed to be guided (Figure 1). In *O. ventralis *(and its congeners) two blotches each are found on the tassels at the tips of the paired pelvic fins, which are conspicuously elongated (Figure 3v, w). Spawning in *O. ventralis *takes place in huge sand bowers of a diameter of up to half a meter in size, which are built by the territorial males in order to attract females. An interested female lays a few eggs in the center of the bower and picks them up into her mouth, after which the male displays its ventral fins with their yellow markings at the egg-laying spot. The female takes up the tassels into her mouth. The tips of the pelvic fins are put into close proximity to the male's genital opening, which discharges sperm [14].

Here, we provide evidence that the same gene is expressed in both kinds of egg-dummies. Just as in the haplochromine species examined, *csf1ra *is expressed in the yellow blotches on the tassels at the tips of the paired ventral fins of males of *O. ventralis*, whereas females do not show *csf1ra *expression (Figure 3y, z). This observation primarily indicates that both kinds of egg dummies are made up of xanthophores, for which *csf1ra *is a good marker gene. The two kinds of independently evolved egg dummies might, in the future, serve as model system to test whether the same genetic pathways are involved in the morphogenesis of a complex ethomorphological trait.

## Methods

### Cichlid fish

Cichlids for *in situ *hybridizations and molecular evolutionary analyses were reared in the Tierforschungsanlage at the University of Konstanz, Germany under standard conditions (12 h light/12 h dark; 26°C). Additional cichlid species were collected in East Africa in autumn 2004. We included representative species of the main haplochromine lineages [7]: *Astatotilapia burtoni *(Lake Tanganyika and surrounding rivers), *Astatoreochromis alluaudi *(lakes and rivers of the Lake Victoria region), *Cyrtocara moorii *(Lake Malawi), *Haplochromis bloyeti *(East African rivers), *Haplochromis obliquidens *(Lake Victoria), *Pseudotropheus *sp. 'bicolor' (Lake Malawi), *Pseudocrenilabrus multicolor *(East African lakes and rivers; Nile system), *Thoracochromis brauschi *(Congo drainage), and *Xystichromis phytophagus *(Lake Kanyaboli; belongs to the Lake Victoria region superflock) (Table 1). The non-haplochromine representatives were: *Altolamprologus calvus*, *Bathybates minor*, *Limnochromis auritus*, *Ophthalmotilapia ventralis*, *Oreochromis tanganicae*, *Plecodus straeleni*, *Telmatochromis bifrenatus*, *Trematocara unimaculatum*, and *Xenotilapia spiloptera *(all belong to the Lake Tanganyika species assemblage), and *Lamprologus teugelsi *(Congo drainage) (see [44] for taxonomic information, and [7,8,22], and this study for phylogenetic assignments). The animals were anesthetized with MS222 (Sigma, Deisenhofen, Germany) prior to manipulations.

**Table 1:** List of specimens used in this study and taxonomic information. The tribe names follow the nomenclature of [57,58]. Note that the 'modern haplochromines' are a monophyletic subgroup of the haplochromine cichlids and contain the entire species flocks of Lake Malawi and the Victoria region, the Tanganyikan Tropheini, as well as some riverine and lacustrine haplochromines in East Africa [7]. Species that exhibit egg-dummies on male anal fins (AF) or pelvic fins (PF) are indicated.

			**Egg-spots**	
			
**Species name**	**Geographic origin**	**Tribe/taxonomic information**	**AF**	**PF**
*Altolamprologus calvus*	Lake Tanganyika	Lamprologini		
*Astatoreochromis alluaudi*	East Africa, Lake Victoria region	Haplochromini	X	
*Astatotilapia burtoni*	Lake Tanganyika area	Haplochromini/'modern haplochromines'	X	
*Bathybates minor*	Lake Tanganyika	Bathybatini		
*Cyrtocara moorii*	Lake Malawi	Haplochromini/'modern haplochromines'	*	
*Haplochromis bloyeti*	East Africa, rivers	Haplochromini/'modern haplochromines'	X	
*Haplochromis obliquidens*	Lake Victoria	Haplochromini/'modern haplochromines'	X	
*Lamprologus teugelsi*	Congo River	Lamprologini		
*Limnochromis auritus*	Lake Tanganyika	Limnochromini		
*Ophthalmotilapia ventralis*	Lake Tanganyika	Ectodini		X
*Oreochromis tanganicae*	Lake Tanganyika	Tilapiini		
*Plecodus straeleni*	Lake Tanganyika	Perissodini		
*Pseudocrenilabrus multicolor*	East Africa, rivers and lakes	Haplochromini	X	
*Telmatochromis bifrenatus*	Lake Tanganyika	Lamprologini		
*Pseudotrophus *sp. 'bicolor'	Lake Malawi	Haplochromini/'modern haplochromines'	X	
*Thoracochromis brauschi*	Congo River	Haplochromini/CSA clade**	X	
*Trematocara unimaculatum*	Lake Tanganyika	Trematocarini		
*Xenotilapia spiloptera*	Lake Tanganyika	Ectodini		
*Xystichromis phytophagus*	Lake Kanyaboli, Lake Victoria	Haplochromini/'modern haplochromines'	X	

*This species from Lake Malawi is a haplochromine representative lacking anal fin egg-spots.

**CSA clade, Congolese/South African clade (according to [7]).

### Fluorescence visualization of xanthophores

For fluorescence visualization of xanthophores in cichlid fins, we used a modified version of the method described in [45]. Amputated anal, dorsal and ventral fins of haplochromine cichlids were mounted in 5% methylcellulose (500 μl) with dilute ammonia (25 μl), and β-mercaptoethanol, pH 10 (1 μl). Digital images were taken with a Zeiss AxioCam Mrc digital camera using a Zeiss Axioplan2 stereomicroscope (Zeiss, Jena, Germany).

### Reverse transcriptase mediated PCR

Prior to *in situ *hybridization experiments, we confirmed expression of *csf1ra *in the haplochromine egg-spots by means of a reverse transcriptase mediated PCR. We amplified and sequenced a 540-bp fragment of *csf1ra *from cDNA that was transcribed from mRNA extracted from *A. burtoni *egg-spot tissue, using the primers F_1986 5'-GCTGCCCTACAATGAAAAGTG-3' and R_2186 5'-TTGACGATGTTCTGGTGGTGA-3'.

### *In situ *hybridization experiments

A 1 233-bp fragment of *csf1ra *was amplified by PCR from *A. burtoni *cDNA using primers F_1986 5'-GCTGCCCTACAATGAAAAGTG-3' and R_3199 5'-AYTGRTAGTTRTTGGKCTTCA-3'. The amplified fragment was cloned into the pCRII vector using the TA Cloning Dual Promoter Kit (Invitrogen, Karlsruhe, Germany). The orientation of ligated inserts with respect to Sp6 and T7 promoters was determined by direct sequencing on an ABI 3100 capillary sequencer using the BigDye terminator reaction chemistry (Applied Biosystems, Darmstadt, Germany). For *in situ *hybridization experiments, DIG-labeled (Roche) antisense RNA was transcribed from a linearized fragment using DIG-labeled dNTPs.

Four rounds of *in situ *hybridization experiments were performed. In total, five male anal fins (plus two female anal fins) were studied from *P*. sp. 'bicolor' and seven male anal fins (plus one female and one dorsal fin) from *A. burtoni*. Furthermore, we used six anal fins (plus one dorsal fin) from *P. multicolor*, three male anal fins from *T. brauschi*, and three male plus two female ventral fins from *O. ventralis*. Amputated fins were fixed in 4% paraformaldehyde in phosphate buffer saline (PFA/PBS) at 4°C overnight, washed twice in PBTw (0.1% Tween-20 in PBS, 0.01 DEPC), and stored in 100% methanol. Then, fins were rehydrated (PBTw washed and post fixed in 4% PFA/PBS) and treated with proteinase *K *(Roche) for 10 min at a final concentration of 14 μg/ml (see [46]). After PBTw washing, cichlid fins were prehybridized (50% formamide, 5 × SSC, 1 mg/ml tRNA, 50 μg/ml heparin, 0.1 % Tween-20, 9 mM citric acid, pH 6.0) and hybridized at 69°C overnight in hybridization buffer plus 1/10 volume of labeled probe. Fins were gradually transferred to PBTw and blocking solution (Boehringer Mannheim, Mannheim, Germany). Anti-(DIG-AP) antibody (Roche) in 0.5% blocking solution and BCIP/X-phos (Roche) were used to visualize target RNA. The tissue was finally fixed in 4% PFA/PBS and stored in 70% glycerol/PBS. As negative control, we applied labeled sense RNA to male fins of *A. burtoni *and *P*. sp. 'bicolor'. Photos of live fins and stained tissue were taken with a Zeiss AxioCam Mrc digital camera using a Zeiss Axioplan2 and Stemi SV11 APO stereomicroscopes. Photos were processed with AxioVision 3.1 (Zeiss) and Photoshop 7.0 (Adobe) software; the background of images was modified with Photoshop 7.0 (Adobe, San Jose, California, USA).

### Polymerase chain reaction and DNA sequencing: the *csf1ra *locus

For PCR amplification of genomic DNA of 18 East African cichlid species, we used the primers listed in Table 2 that had been designed against a reference sequence from *A. burtoni *(GenBank accession number: DQ386648; [24]). We also resequenced the *A. burtoni *locus with these primers. Exon1 and the relatively large introns 1, 16, and 19 were not sequenced. The primers for amplification of the upstream region of *csf1ra *were 26M7_3F 5'-CTCACCTCTGCGGATGTTTC-3' and 26M7_3R 5'-GCCACAGCATAAGGAAGGAC-3'. For the determination of the cDNA sequence of *csf1ra *from a normalized library ("pinky") made from different *A. burtoni *mRNA pools [47], we additionally used the primers listed in Table 3. For comparative analyses, we also determined, for the same set of species, two segments of another type III receptor tyrosine kinase, *kita*. This pigmentation gene plays an important role in melanophore development [31]. It is therefore another candidate for being an important color gene in cichlid fishes. We determined a ca. 300 bp segment in the extracellular domain of *kita *(including the second immunoglobulin domain) using primers Burt-Kit-F-474 5'-GATCTGGAGAATATGCACCTGGA-3' and Burt-Kit-R-672 5'-ATCACTCTTGTGGATGGTTGGAG-3' and a ca. 500 bp segment corresponding to the kinase insert domain using primers a Burt-Kit-F-2023 5'-TATTGTCAACCTACTGGGAGC C-3' and Burt-Kit-R-2316 5'-AACCGTCATCAGCAAACATCTC-3'. PCR reactions were carried out under standard conditions on ABI 9700 thermocyclers (Applied Biosystems). PCR products were purified with Qiaquick spin columns (Qiagen, Hilden, Germany). Sequencing reactions were performed with forward and reverse primers (see above for details) using the BigDye terminator reaction chemistry (Applied Biosystems). DNA sequences were detected on ABI 3100 automated capillary DNA sequencers (Applied Biosystems).

**Table 2:** Primers used for sequencing of the *csf1ra *locus in 19 cichlid specimens

**Primer**	**Sequence**
ex2_3F	5'-AAAACCCTCAGAGACCATCAG-3'
ex2_3R	5'-TCCAATAAACGCATCAGAGAG-3'
ex4_6F	5'-GGTAGAGCAGGTGGTTCAGTC-3'
ex4_6R	5'-CAGCACTCTTCCCTCTTTGAG-3'
ex7_9F	5'-GAAAAGTGCGAAAATCAAGTG-3'
ex7_9R	5'-TCAGAAGGGAAGTAAAGGGC-3'
ex10_11F	5'-ATTACTGGATGTGGTTCAGATC-3'
ex10_11R	5'-ACAGAGGAGCATTTTGACTTC-3'
ex13_16F	5'-TTTTTGAGTTGAGCGTGACAG-3'
ex13_16R	5'-AACCTCACACCCATCCTGC-3'
ex17_19F	5'-TCTTATTGTCTTTCACTGGGC-3'
ex17_19R	5'-ATCAGTCGTAAAAACTCTGCTG-3'
F_1986	5'-GCTGCCCTACAATGAAAAGTG-3'
R_2186	5'-TTGACGATGTTCTGGTGGTGA-3'
F_2961	5'-ARATGTGCTGGAAYCTGGA-3'
R_3199	5'-AYTGRTAGTTRTTGGKCTTCA-3'
F_1962	5'-ACTACACCTTYRTYGACCCCAC-3'
R_2269	5'-CARRAAGTTSAGCAGGTCGCC-3'
F_1758	5'-CCCACATCACAAAGCACAGA-3'
R_2425	5'-GCCAGTGGGAAGAAATGAG-3'
F_0732	5'-TAAGGCCTTTTCCATCAATG-3'
R_1417	5'-GCGATAGTCAGTGTGCTCTC-3'

**Table 3:** Primers used for cDNA amplification and sequencing

**Primer**	**Sequence**
10F_ex1	5'-TACCTTATGCTGTGGCTTGTG-3'
1557F_ex2	5'-GATCTGAGGTGTGAGGGTGAA-3'
4134F_ex9	5'-GAGTACGGGGCTGTGGAAGTG-3'
7315F_ex17	5'-AGAGATGTGGCTGCGAGGATT-3'
3650R_ex7	5'-TGCGTTGGTCATTTCACTCTG-3'
6030R_ex16	5'-CTTGGAAGGAAAATCTGAGCA-3'

### Molecular evolutionary analyses: the *csf1ra *locus

DNA sequences were quality trimmed with Phred [48] and assembled with Sequencher 3.0 [49]. Sequences have been deposited in GenBank under the accession numbers EU042675–EU042749 (*csf1ra*) and EU042637–EU042674 (*kita*) (note that the segment spanning exons 17 to 19 in *csf1ra *could not be amplified for *T. unimaulatum*). Exon/intron boundaries were identified using a reference sequence from *A. burtoni *(DQ386648) and checked by homology comparison with reference sequences from pufferfish (U63926), zebrafish (AF240639) and trout (AJ417832). The protein structure of Csf1ra and the functional domains were identified from homologous sequence motifs in zebrafish (using the Protein families database Pfam [50]) and pufferfish [23]. MatInspector from Genomatix [51] was used to identify putative promoter modules in the upstream regions of *csf1ra*.

Maximum likelihood and maximum parsimony phylogenetic analyses with 19 cichlid taxa were performed with Paup * 4.0b10 [52]. The appropriate model parameters for the maximum likelihood analysis were determined by means of a likelihood ratio test with Modeltest version 3.6 [53]. For the maximum likelihood tree search based on the non-coding section of the *csf1ra *locus (4 171 bp), we used the general time reversible model of molecular evolution (six types of substitutions) with a proportion of invariable sites of 0.4714 and a gamma substitution correction (α = 0.8669). An unweighted heuristic maximum parsimony search was performed with the same dataset (50 replicates). Bootstrap analyses were performed with 100 replicates under the maximum likelihood criterion and with 1 000 replicates for maximum parsimony analysis. Alternative branching orders at critical branches within the haplochromines were evaluated by means of a nonparametric Shimodaira-Hasegawa test under a resampling-estimated log likelihood with 1 000 bootstrap replicates as implemented in Paup*: The optimal maximum likelihood topology with *Pseudocrenilabrus multicolor *as sistergroup to *Thoracochromis brauschi *and all remaining haplochromines was tested against trees in which *T. brauschi *or *Astatoreochromis alluaudi *were forced to occupy the most ancestral position within the haplochromines (see also [7]).

Sliding window analyses for calculating the nucleotide diversity (π) in the coding region of *csf1ra *in haplochromines *versus *non-haplochromines were performed with DNASP 4.0 [30]. We used a window size of 50 bp and an overlap of 10 bp. We also used DNASP for the calculation of the *d*N/*d*S ratio in haplochromines compared to non-haplochromines using the same 50-bp windows (overlap: 10 bp). We then used HyPhy [54] for the reconstruction of *d*N/*d*S ratios, based on maximum likelihood, on the branches of the phylogeny obtained before (see above), analyzing the entire dataset as well as the first 1 551 bp only, which correspond to the extracellular ligand-binding domain of *csf1ra*. We applied a site-to-site variation model with two independent gamma distributions and the MG94 model, and tested for relevant internal branches in the tree. The reconstructed *d*N/*d*S ratios were visualized in form of a branch-scaled tree applying the Suzuki-Gojobori derived adaptive selection tool implemented in HyPhy. We also used DNASP for sliding window analyses in *kita *and HyPhy to plot *d*N/*d*S ratios for the two segments of *kita *on the maximum likelihood tree.

### Character state reconstructions

The characteristic egg-dummies of the haplochromines have been identified as a potential key evolutionary innovation that might be directly related to the evolutionary success of this most species-rich group of cichlid fishes [7] (but see [55]). As some ancestral (but also some derived) species of haplochromines do not show egg-dummies on male anal fins, we intended to reconstruct the evolutionary origin of these markings based on the new phylogenetic and comparative morphological data now available. Specifically, we wanted to evaluate the hypothesis that these characteristic egg-spots evolved only once in the ancestor of the haplochromines and that the missing ovoid markings on anal fins of males of some species are due to secondary loss. We used Mesquite 1.03 [56] for maximum likelihood and maximum parsimony ancestral state reconstructions of the evolutionary origin of egg-dummies on male anal fins, on the basis of a consensus phylogeny of East African cichlid fishes. We also mapped the evolutionary origin of the characteristic polygynous mating system with maternal mouthbrooding involving egg-spots on that phylogeny. The consensus tree was built using a previous mitochondrial phylogeny [7] as well as the present phylogenetic hypothesis that is based on nuclear DNA. Note that the ancestral polytomy between the *Pseudocrenilabrus*, the *Astatoreochromis*, the Congolese/South African lineage, and the modern haplochromines [7] remained unresolved in all available phylogenetic hypotheses.

## Authors' contributions

WS, IB and AM designed the study. WS and IB carried out the molecular work and the analyses. All authors contributed to the preparation of the manuscript, and read and approved the final version.

## Supplementary Material

Additional file 1 **The breeding cycle of *Astatotilapia burtoni***. Modified from [59]. Click here for file

Additional file 2 **Amino acid substitutions in Csf1ra in haplochromines**. The numbering of amino acids is relative to the start site in *A. burtoni *(DQ386648). Click here for file

Additional file 3 ***d*N/*d*S ratio of haplochromines compared to non-haplochromines in two segments of *kita***. The sliding window analysis with DNASP did not detect a dN/dS > 1 in the extracellular domain (a) or in the intracellular domain (b) of *kita*. See Figure 4b for the same analysis in *csf1ra* and the Methods section for details of the analysis. Click here for file
