# Supplementary figures and images for: Adaptive sequence evolution in a color gene involved in the formation of the characteristic egg-dummies of male haplochromine cichlid fishes
Source: BMC Biol. 2007 Nov 15;5:51. doi: 10.1186/1741-7007-5-51 (PMC2254590; doi:10.1186/1741-7007-5-51)

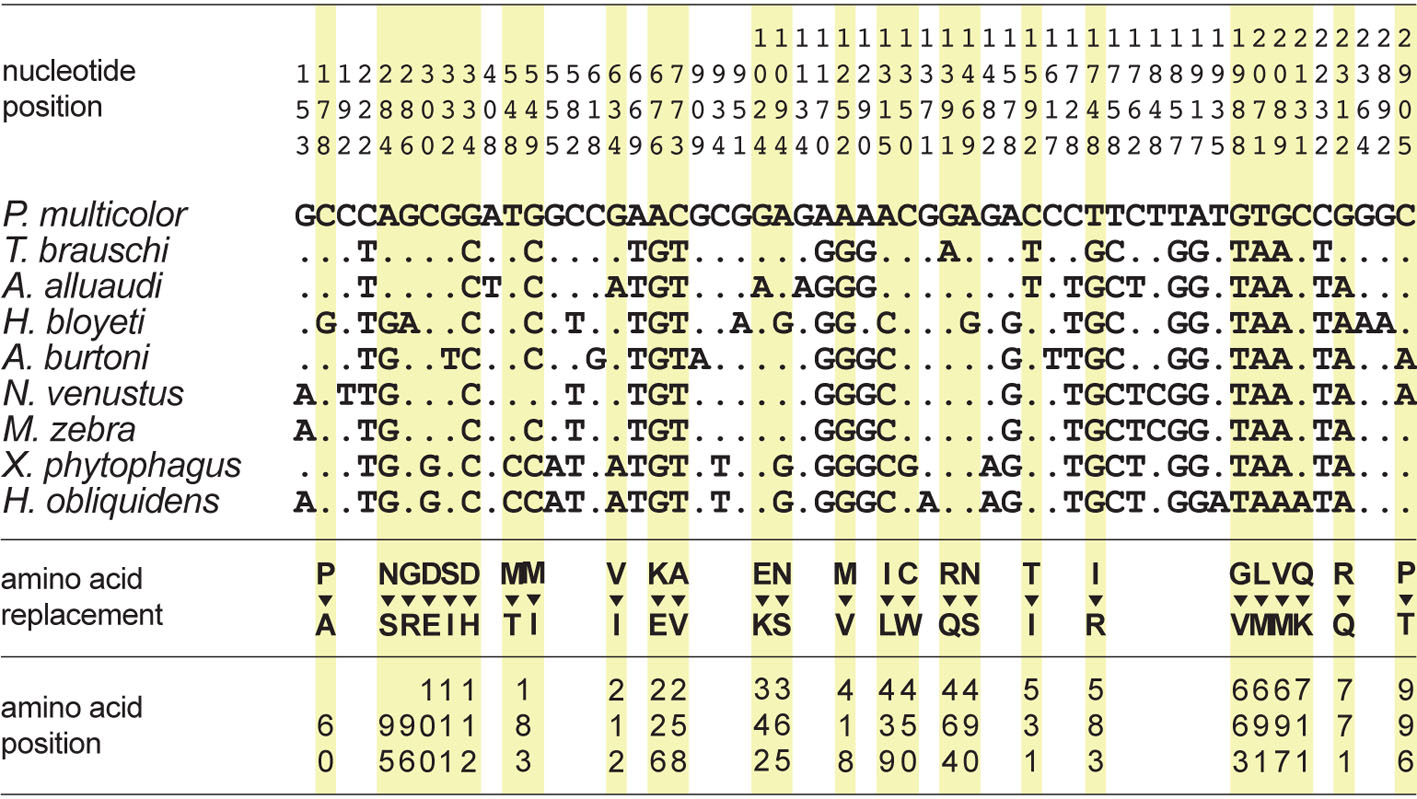

Supplement: Additional file 2 — Amino acid substitutions in Csf1ra in haplochromines. The numbering of amino acids is relative to the start site in A. burtoni (DQ386648). [file 1741-7007-5-51-S2.jpeg]

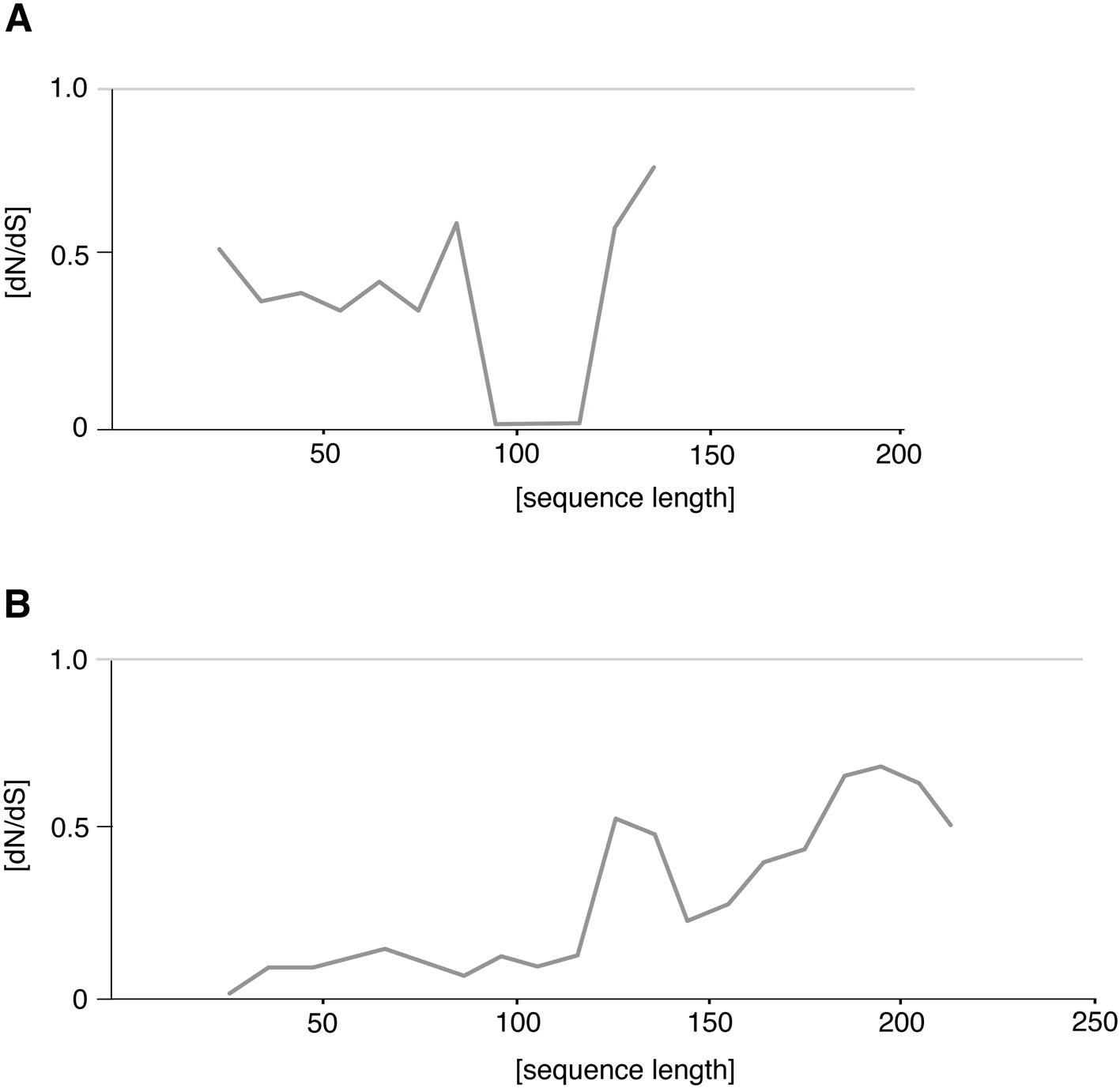

Supplement: Additional file 3 — dN/dS ratio of haplochromines compared to non-haplochromines in two segments of kita. The sliding window analysis with DNASP did not detect a dN/dS > 1 in the extracellular domain (a) or in the intracellular domain (b) of kita. See Figure 4b for the same analysis in csf1ra and the Methods section for details of the analysis. [file 1741-7007-5-51-S3.jpeg]
